# Impact of diazotrophs on marine food webs and the biological carbon pump: progress and remaining challenges

**DOI:** 10.1093/ismejo/wraf291

**Published:** 2026-01-06

**Authors:** Sophie Bonnet, Hugo Berthelot, Ilana Berman-Frank

**Affiliations:** Aix Marseille Université CNRS/INSU, Université de Toulon, IRD, Mediterranean Institute of Oceanography (MIO), 13009 Marseille, France; IFREMER, DYNECO, Pelagos Laboratory, 29280 Plouzané, France; Department of Marine Biology, Leon H. Charney School of Marine Sciences, University of Haifa, Haifa, Israel

**Keywords:** marine diazotrophs, nitrogen fixation, marine food webs, biological carbon pump

## Abstract

Marine diazotrophs are microscopic planktonic organisms ubiquitous in the ocean, that play a major ecological role: they supply nitrogen to the surface ocean biosphere, an essential but scarce nutrient in ~60% of the global ocean. Over the past decades, they have attracted considerable attention, with numerous studies providing key insights into their diversity, lifestyle, biogeographical distribution, and biogeochemical role in planktonic ecosystems. An increasing number of studies show that these microbes regulate marine productivity and shape the food web by alleviating nitrogen limitation, thereby contributing to carbon sequestration to the deep ocean. Yet, the diazotroph-derived organic carbon exported to the deep ocean is still poorly quantified, limiting robust estimates of the ocean’s contribution to CO₂ sequestration and climate change mitigation under present and future conditions. This knowledge gap reflects the complexity of diazotroph export pathways to the deep ocean, whose quantification and variability drivers remain difficult to resolve with current methods. This review aims to synthesize current knowledge on the role of diazotrophs in their interactions with the food web and the biological carbon pump (BCP), reanalyze existing datasets, identify key knowledge gaps, and propose future research directions.

## Introduction

Diazotrophs are microscopic organisms widespread in the ocean that play a crucial ecological role by supplying nitrogen (N) to the surface ocean biosphere, an essential yet scarce nutrient in most of the world’s surface waters [1]. Diazotrophs convert atmospheric N₂ -metabolically unavailable to most organisms- into ammonia, a readily accessible form of nitrogen, through a process known as N₂ fixation. This source of newly introduced N sustains CO_2_ fixation by phytoplankton into organic carbon (OC), which in turn, sustains the food web and OC transfer (i.e. export) and sequestration at depth through the so called “N_2_-primed prokaryotic C pump” [2].

The number of studies on planktonic marine N_2_ fixation (i.e. by organisms suspended in the water column) has increased exponentially over the past three decades, providing major insights into the diversity of diazotrophs, their lifestyles and their geographical distribution [3, 4]. Marine diazotrophy was historically believed to be dominated by the large filamentous cyanobacteria *Trichodesmium* and *Richelia* mostly thriving in (sub) tropical waters [5, 6]. High-throughput nucleic acid sequencing has since then revealed an unexpected diversity of diazotrophs including unicellular cyanobacteria (UCYN) living under various lifestyles (i.e. free-living [7, 8], as symbionts [9] or even as cellular organelles of eukaryotes [10]). Many lineages of heterotrophic or photoheterotrophic bacteria (called Non-Cyanobacterial Diazotrophs, NCDs) [11, 12] have also been described, spanning all ocean depths as free-living, particle-associated and symbionts of eukaryotic algae [13] and larger organisms [14]. Gradually, these discoveries revealed that the distribution range of planktonic diazotrophs extended beyond the tropics and the photic layer, and diazotrophs are reported in temperate [15, 16], high latitude [17, 18], coastal [19] and aphotic [20–22] waters, with the different groups having distinct biogeographic niches [15, 23–25], [17, 18, 26–28].

Current data show that diazotrophs inhabit all oceans; however, our knowledge of their biogeochemical impact on the marine food web and the biological carbon pump (BCP) remains limited by significant knowledge gaps. These gaps hinder accurate representation of diazotrophs and their fate in biogeochemical models, resulting in substantial uncertainties in the projections of future net primary production [29] and carbon export fluxes [30]. In this review, we outline our current understanding and reanalyze some existing datasets focusing on two major questions: (i) How do diazotrophs interact with the food web?, (ii) What is the role and contribution of diazotrophs to the BCP? For each, we also identify knowledge gaps and suggest future directions (Table 1). A deep understanding of these questions is critical, as diazotrophs currently sustain the majority of new primary productivity in (sub)tropical oceans [31, 32] and are likely to assume an even more prominent role under future climate-driven expansions of oligotrophic regions [33, 34].

**Table 1:** Knowledge gaps and suggested future directions of research on the role of diazotrophs in the marine food web and the biological carbon pump.

Domain	Knowledge gaps	Future directions
Diazosphere micro-environment	Poorly constrained chemical communication and metabolite fluxes; role of epibionts in nutrient cycling and trace metal acquisition.	Characterize microbial consortia with meta-omics; quantify chemical gradients and cell–cell signaling; identify mutualistic interactions (e.g. Fe ligands, DMSP).
Interactome (plankton associations)	Co-occurrence networks suggest links but causality and trophic nature are unresolved.	Combine process experiments on species interactions, larger datasets, long-term isotope tracing and molecular markers to confirm trophic links.
Trophic transfer of DDN	Efficiency of transfer across trophic levels poorly constrained; role of fecal pellets as export vectors not quantified.	Trace DDN into specific functional groups (diatoms, picophytoplankton, zooplankton) with isotope labeling; measure incorporation into fecal pellets and sinking flux.
Direct export of diazotrophs	Sinking velocities, remineralization rates, and fate in mesopelagic remain largely unknown; variability among taxa underexplored.	Conduct *in situ* measurements with sediment traps, Marine Snow Catchers, and autonomous platforms; quantify turnover rates and ballasting effects (dust, biominerals).
Indirect export via consumption	Magnitude and efficiency of secondary export pathways uncertain; contribution of zooplankton-mediated packaging (fecal pellets, aggregates) not assessed.	Quantify indirect fluxes across ecosystems and seasons; couple ^15^N tracer experiments with zooplankton gut content and pellet analyses.
Biogeochemical modeling	Current models do not represent diazotroph-mediated export and differential fate of taxa; uncertainties in parameterization of sinking, remineralization, and DDN transfer.	Explicitly represent functional groups of diazotrophs; incorporate particle size, density, and sinking dynamics; integrate field-based constraints into global models.
Observational strategies	Spatial/temporal resolution of diazotroph export is too low; lack of concurrent surface–deep ocean observations.	Develop high-frequency autonomous platforms (gliders, floats, moorings) with sensors for N_2_ fixation, particle flux, and plankton community composition.

### (1) Diazotroph interactions with the food web

Planktonic diazotrophs, like all plankton organisms, engage in continuous and dynamic interactions with one another. By providing a source of new bioavailable N (hereafter called Diazotroph-Derived N, DDN) to ecosystems often limited in N, diazotrophs structure the food web, which subsequently impacts biogeochemical cycles [35]. Interactions occur at multiple levels and can be categorized based on (i) cellular proximity: intracellular, epicellular, or extracellular, and (ii) the nature of the interaction: symbioses, close physical associations within the microenvironment surrounding the diazotroph (the “diazosphere”), or co-occurrence and trophic links within the same environment (the “interactome”).

### Symbioses

For centuries, agriculture relied on multiple strategies to supply soil with nitrogen, including diazotroph-plant symbioses in legumes, as well as organic inputs such as manure and guano. These practices provided essential nitrogen for crop growth until the advent of the Haber-Bosch process enabled large-scale industrial nitrogen fixation. Such tight diazotroph-plants interactions are also a common feature in the ocean, with accumulating examples of diazotrophs providing N to their hosts. The most iconic associations are probably the Diatom Diazotrophs Associations (DDAs), such as the heterocyst-forming *Richelia intracellularis* in association with the diatoms *Rhizosolenia* spp. or *Hemiaulus* spp., which are widespread in subtropical oceans [36]. The filamentous diazotroph *Calothrix* in episymbiosis with the diatom *Chaetoceros* spp. or UCYN with the diatom *Climacodium* spp. are also commonly observed [36–38]. They are both easily detectable by epifluorescence microscopy due to their large size (>10 μm) and richness in phycoerythrin. Recently, new DDAs were described involving (i) non photo-pigmented cyanobacteria phylogenetically close to UCYN-C and pennate diatoms of the *Rhopalodiaceae* family, and recovered both in the water column [39] and in sediment traps [40], (ii) non-cyanobacterial diazotrophs belonging to *Rhizobiales* (*Candidatus* Tectiglobus diatomicola), living in endosymbiosis with a diatom host, *Haslea* spp. [13]. These potentially widespread associations have been overlooked due to the absence of photosynthetic pigments in diazotrophs, hindering their microscopic detection.

The non-pigmented UCYN-A, long described as living in obligatory symbiosis with its haptophyte host [7, 41], is now recognized as an early evolutionary stage N_2_-fixing organelle, or “nitroplast” [10]. This association is suspected to contribute to a great share of the oceanic N_2_ fixation budget [42].

Initial research of these symbiotic interactions between diazotrophs and eukaryotes hypothesized that provision of N from the diazotrophs to the host cell drives the association. This has been demonstrated by using stable isotope probing and isotope mapping with nanoSIMS on pigmented DDAs [43], and soon after on UCYN-A associations [41, 44]. In non-photoautotrophic diazotrophs, OC derived from photosynthesis by the host is given in exchange for fixed N, which has been described for UCYN-A [41] and the Rhizobia-diatom associations [13].

**Figure 1:**
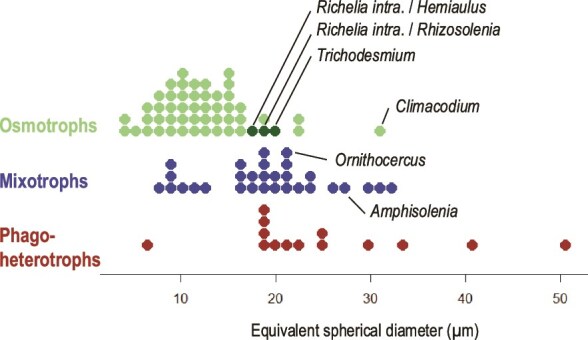
Relationship between trophic strategy and organism (microbial) size represented by the median equivalent spherical diameter of organisms found at low nitrate concentrations stations (<0.05 μM) during the Tara Ocean Expedition. Trophic strategy (pure osmotrophs, mixotrophs and phagoheterotroph) is defined for the 5–20 μm and 20–180 μm size fractions. Diazotrophs (Trichodesmium, Richelia intra. associated with Hemiaulus or Rhizosolenia) appear in the upper size range of pure osmotrophs (dark green). Climacodium, Ornithocercus and Amphisolenia are known (or suspected) to host symbiotic cyanobacterial diazotrophs.

The phylogenetic diversity of diazotrophs in symbiosis with protists suggests a co-evolution process [10, 45], which could explain the competitive advantage of such an association. In nutrient-depleted environments, the growth of osmotrophs (organism that absorbs nutrients from solution) is limited by the diffusive transport of ionic solutes (nutrients dissolved in seawater) through their cellular membrane. This tends to favor small cells having high surface to volume ratios. This limitation does not apply to diazotrophs for the acquisition of N because dissolved N_2_ concentrations (>400 μM) are several orders of magnitudes higher than all the other N sources bioavailable (generally between 0 and 7 μM [46]). Being associated with a diazotroph allows relatively large protists to meet their N needs. To illustrate this, we extracted the annotated plankton images (from 5–20 μm and 20–180 μm size fractions) from the Tara Ocean database [47] at stations where surface NO_3_^−^ concentrations were below the detection limit of instruments (<0.05 μM, therefore likely N-limited regions). We classified organisms into three trophic strategies: osmotrophs (absorbing dissolved nutrients), phagoheterotrophs (ingesting particulate prey such as bacteria or detritus), and mixotrophs (combining autotrophic nutrition via osmotrophy with heterotrophic nutrition via osmotrophy or phagoheterotrophy). We then plotted these groups according to their median equivalent spherical diameter (Fig. 1).

The dataset reveals that in the purely osmotrophic group, the largest organisms are mostly represented by diazotrophs (*Trichodesmium* spp. and *Richelia* spp. in symbiosis with *Hemiaulus* spp. or *Rhizosolenia* spp.) (Fig. 1). It also reveals that some of the few larger osmotrophic or mixotrophic groups are known to host diazotrophic symbiont (e.g. the diatom *Climacodium* [37]) or suspected to do so (the dinoflagellates *Amphilozenia* and *Ornithocercus* [48]). By overcoming diffusion-limited transport of ionic N solutes, diazotrophs (in association or not with larger organisms) seem to escape the expected particle size distribution of osmotrophs, which in turn has the potential to affect the structure of the food web and organic matter export (as discussed in Section 2).

### Diazosphere: an attractive micro-environment

In addition to species-specific symbiotic associations, diazotrophs form specific microenvironments, referred here as the diazosphere, defined as the microzone surrounding individual cells or colonies (typically 100–1000 μm in size). Within this diazosphere, active and diverse consortia of epibionts develop including viruses, bacteria (heterotrophs and cyanobacteria), protists (fungi, diatoms, dinoflagellates, chrysophytes, ciliates, amoebae), nauplii and juveniles of metazoans [49]. The bacterial community inhabiting the diazosphere is generally less diverse than the surrounding bacterioplankton [50] and plays a key role in regulating the cycling of C, N, phosphorus, iron, and vitamin B_12_ within this microenvironment [51].

Bacteria associated with diazotrophs exhibit upregulated transcript activity of N-cycling genes involved in both assimilatory and dissimilatory processes, confirming that N fluxes are key processes in the diazosphere [52, 53]. A comprehensive study on N fluxes within *Trichodesmium* colonies showed that these microsystems are characterized by net gain of N and high recycling processes rather than N removal processes (denitrification), the latter being hindered by high oxygen within the colonies [54]. Moreover, NH_4_^+^ concentrations measured in the center of the colonies were 6-fold higher than NH_4_^+^ in the ambient seawater [54].

Outside the open ocean, in brackish waters and saline lakes, bacterial epibionts of *Nodularia* colonies (a filamentous diazotrophic cyanobacterium) directly assimilate freshly produced DDN [55]. The capacity to sense and swim toward the colonies might thus confer a decisive recruitment advantage for planktonic cells in environments where these diazospheres are likely to occur. Chemotaxis might be promoted by the production of dimethylsulfoniopropionate (DMSP) -a strong chemoattractant- by *Trichodesmium* [51]. In turn, *Trichodesmium* may benefit from the iron-complexing molecules produced by heterotrophic bacteria which facilitate accessibility to iron [56, 57]. The characterization of chemical communication pathways at the intra- and inter-species levels is still at its infancy. The few examples described to date account probably for only a small fraction of the cell-to-cell signaling network leaving a large knowledge gap regarding communication within and outside these close associations.

### Diazotroph interactome and trophic links

When examining the water column scale, several studies have suggested successions and/or links between diazotroph blooms and surrounding non-diazotrophic communities.

*Diazotroph interactome*. Devassy *et al.* [58] provided the first evidence that the decline of a *Trichodesmium* bloom was accompanied by an increase in diatom abundances, followed by a sequential proliferation of cladocerans, dinoflagellates, green algae, and ultimately copepods. Subsequent studies further confirmed shifts in non-diazotrophic plankton communities during and after bloom events: for instance, elevated abundances of non-diazotrophic phytoplankton -including the harmful dinoflagellate *Karenia brevis-* were recorded in the Gulf of Mexico following *Trichodesmium* blooms [59]. Other studies have also reported recurrent associations between diazotrophs and diatoms [60–62], as well as with dinoflagellates [63].

**Figure 2:**
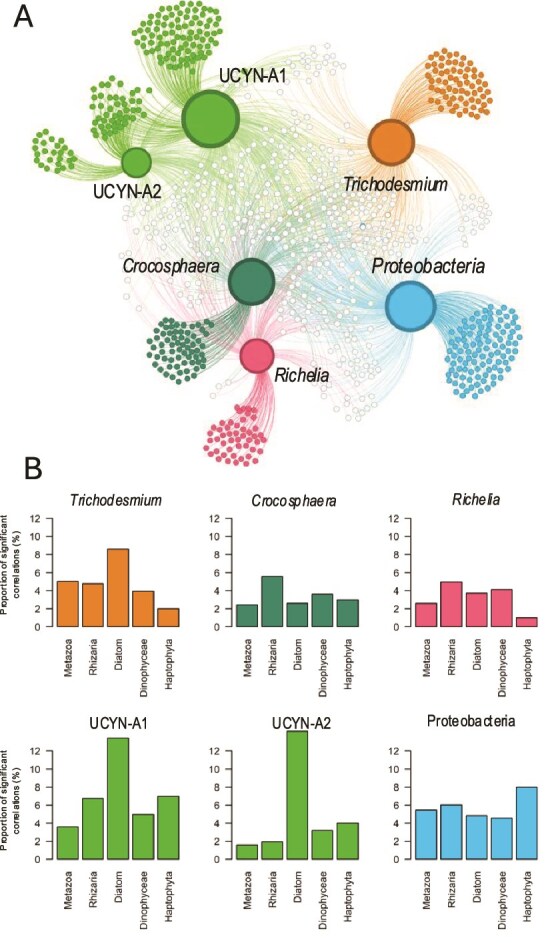
(A) Integrated plankton community network based on prokaryotic diazotrophs (detected by shotgun sequencing of *nifH*) and eukaryotes (detected by amplicon sequencing of the 18SV4 rRNA gene) from Tara Oceans samples, restricted to stations with NO₃^−^ < 0.05 μM. Large nodes represent diazotrophs, with node size proportional to the number of positive correlations with non-diazotrophs. Small nodes represent taxa correlated with at least one diazotroph. Filled nodes indicate ASVs (amplicon sequence variant) correlated with a single group of diazotrophs, whereas open nodes indicate ASVs correlated with two or more groups. (B) Proportion of significant (*P <* .0001) ASV–diazotroph correlations relative to the total ASVs detected in key planktonic groups (Metazoa, Rhizaria, diatoms, Dinophyceae, Haptophyta).

To explore the diazotroph interactome, i.e. eukaryotic plankton groups co-occurring with diazotrophs, we delved into the Tara Ocean genes dataset to test significant correlations between the presence of diazotrophs and eukaryotes in the global ocean (Fig. 2, Table S2). Even tough co-occurrence does not necessarily imply direct trophic or symbiotic interactions, it provides valuable insights into potential ecological associations and serves as a basis for exploring microbial interactions within complex communities. We correlated the diazotroph abundances using the *nifH* genes abundances retrieved from metagenomic shotgun sequencing [36], with the eukaryotes’ abundances using ASVs from the amplicon sequencing of the V4 region of the 18S rRNA gene [64] (see note on Methods (1)). We selected samples from stations with ambient NO_3_^−^ concentrations below detection limit (0.05 μM), where new N provided by diazotrophs will likely influence the planktonic community.

We assessed the performance of the interactome using well-known diazotroph–protist associations. The UCYN-A/*Braarudosphaera* (coccolithophores) symbiosis was clearly detected: UCYN-A1 and UCYN-A2 were the only diazotrophs significantly correlated with *Braarudosphaera* ASVs (*r* = 0.72–0.86, Table S2), representing the strongest associations observed. The *Richelia*–diatom interaction was also recovered, with *Richelia* correlating with the diatoms *Rhizosolenia* (*r* = 0.49, Table S2) and *Chaetoceros* ASVs (Amplicon Sequence Variants), consistent with previous reports [65, 66]. Together, these results validate the approach and demonstrate its ability to capture established -and potentially novel- diazotroph–protist interactions. Given the large number of significant correlations between diazotrophs and eukaryotes (Table S2), we focus below on the main ones, specifically the strongest correlations (*r* > 0.60), to provide a concise summary.

**UCYN-A1** exhibited the highest proportion of significant correlations with eukaryotes, particularly with diatoms (Fig. 2; Table S2). The strongest associations (*r* > 0.60) were observed with *Frustulia, Fragilariopsis, Chaetoceros*, and *Pseudo-nitzschia*. UCYN-A1 also correlated strongly with **Rhizaria** (mainly Radiolarians) and with ***Dinophyceae***, showing numerous strong correlations (*r* > 0.60) with ***Syndiniales***, a group of parasitic marine alveolates. **UCYN-A2** showed an even higher proportion of significant correlations with diatoms, with particularly strong links (*r* > 0.60, Table S2) to *Chaetoceros, Actinocyclus*, and *Pseudo-nitzschia*. Similar to UCYN-A1, UCYN-A2 was also strongly correlated with Dinophyceae, and especially with *Syndiniales*.

***Trichodesmium*** spp. exhibited significant correlations with several diatoms, notably *Meuniera* and *Chaetoceros*, although these associations were weaker than those of UCYN-A and none exceeded *r* = 0.60. In contrast, *Trichodesmium* showed strong correlations (*r* > 0.60) with a limited number of *Dinophyceae*, particularly *Archaeperidinium* and *Alexandrium*, but weaker associations with *Syndiniales* (Fig. 2; Table S2).

**The unicellular diazotrophic cyanobacterium *Crocosphaera* spp.** displayed its highest proportion of significant correlations with Rhizaria (mostly Radiolarians) and with *Dinophyceae*, especially *Gymnodiniales* such as W*arnowia spp*. Correlations with *Syndiniales* were also detected, but were comparatively weaker than with *Gymnodiniales* (Fig. 2; Table S2).

*Richelia* spp. also displayed its highest proportion of significant correlations with **Rhizaria** (mostly Cercozoans and Radiolarians) and with ***Dinophyceae***, mostly *Syndinales* (Fig. 2; Table S2).

*Pseudomonadota (Proteobacteria)*, a major phylum of Gram-negative bacteria- carrying the *nifH* gene showed numerous significant correlations with eukaryotes, especially Haptophytes, although none exceeded *r* = 0.60 (Fig. 2; Table S2). These broad associations likely reflect their wide distribution in the ocean [67]. Their ecology is still poorly understood, but traits such as chemotaxis and a particle-attached lifestyle may allow them to access low-oxygen niches favorable for N₂ fixation [11, 68, 69], likely explaining the high number of interactions compared to cyanobacterial diazotrophs. Their ability to fix N_2_ could provide a strong advantage within the carbohydrate-rich phycosphere, potentially supporting mutualistic associations with phytoplankton through C–N exchanges [70]. Further investigation is needed, as heterotrophic diazotrophs may contribute up to ~10% of global marine N₂ fixation [71].

How do diazotrophs associate with metazoans? *Trichodesmium* exhibits the highest number of significant correlations with metazoans, particularly with copepods of the genera *Miracia, Acartia*, and *Parvocalanus* for the strongest ones (*r* > 0.60) (Fig. 2; Table S2). These correlations contrast with previous studies showing that only a few genera of harpacticoid copepods (notably *Microstella*) feed on *Trichodesmium* [72–74]. Co-occurrence does not provide evidence of a trophic link, but it is likely that a greater number of mesozooplankton species feed on *Trichodesmium*, as shown by the consistent detection of *Trichodesmium* gene sequences in the viscera of calanoid copepods [75, 76]. The strongest metazoan correlations for UCYN-A1, *Crocosphaera*, and *Richelia* were also with copepods. In contrast, Proteobacteria showed their strongest associations with decapods, whereas UCYN-A2 correlated most strongly with urochordates (doliolids), likely reflecting distinct ecological niches compared to the other diazotrophs.

In conclusion, the diazotroph interactome reveals both established and previously unrecognized associations with protists and metazoans, highlighting their key role in structuring plankton communities. Strong correlations between UCYN-A and diatoms, and between *Trichodesmium, Crocosphaera*, or *Richelia* and diverse eukaryotes, provide testable hypotheses for ecological interactions. Future work combining microscopy, single-cell approaches, and experimental studies will be essential to validate these associations and to better assess the ecological role of both cyanobacterial diazotrophs and NCDs in shaping plankton communities and driving marine N and C cycles.

*Diazotroph-Derived N transfer in the food web*. The recurrent co-occurrence of diazotrophic and non-diazotrophic species may indicate potential trophic interactions, yet such links are rarely demonstrated owing to methodological challenges. Stable isotope assays, where ^15^N_2_ is provided to planktonic communities, have explored some trophic links by examining short-term transfer (from a few hours to a few days) of DDN from its fixation and assimilation by diazotrophs to non-diazotrophic planktonic communities. Size fractionation was initially utilized to differentiate diazotrophs from non-diazotrophs, assuming the smallest size fractions did not contain any active diazotroph [77, 78]. Yet, these methods do not distinguish between the transfer of DDN to a specific size fraction and the N₂ fixation carried out by diazotrophs within that same fraction, potentially leading to an overestimation of DDN transfer. To circumvent this limitation, Mulholland *et al.* [79] used dialysis bags to isolate *Trichodesmium* colonies from the other planktonic cells. These experiments pointed out a DDN reincorporation to non-diazotrophs communities accounting for ~11% of the fixed N_2_. This fraction could meet the N demand of the toxic dinoflagellates *Karenia brevis* that often blooms after *Trichodesmium* blooms in the Gulf of Mexico [79].

Overall, these approaches do not identify the planktonic groups (e.g. autotrophic *vs*. heterotrophic, small *vs*. large phytoplankton) that benefit the most from this DDN source, despite their potential to differentially affect the structure of the food web. The development of tools to measure isotopic ratios at the single-cell level (high-resolution nanometer scale secondary ion mass spectrometry, nanoSIMS) has allowed the direct and more accurate quantification of the DDN transfer to the planktonic food web with an unprecedented taxonomic resolution. Several studies combined single-cell ^15^N isotopic analyses using nanoSIMS with cell sorting by flow cytometry [60] and applied those techniques to a broad range of trophic conditions in subtropical waters, involving several configurations: a diazotroph community dominated by *Trichodesmium vs.* one dominated by UCYN [80–82]. Diazotrophs released 7–50% of the recently fixed N (in line with earlier studies [35]), and this DDN was transferred to non-diazotrophic plankton within 24–48 h (both eukaryotic and prokaryotic such as non-diazotrophic picocyanobacteria), ranging from 5 to 21% of total N_2_ fixation [80–82]. In the case of natural *Trichodesmium* blooms, the primary beneficiaries of this DDN were diatoms, whose abundance increased 15-fold (notably *Cylindrotheca closterium*).

Diatoms are efficient exporters of OC to depth [83] and may thus drive secondary export of diazotroph-derived material out of the euphotic zone (see section below). The transfer rate of DDN from UCYN appears to be higher than that of *Trichodesmium*, accounting for ~20% of total N₂ fixation over 48 h. This transfer also markedly influenced the community structure, resulting in significant increases in the abundance of picophytoplankton (*Synechococcus*) and diatoms (*C. closterium*). Among the affected groups, diatoms and picophytoplankton were the primary beneficiaries of UCYN-produced DDN. Following ^15^N_2_ incubations, the NH_4_^+^ pool was highly enriched with ^15^N, suggesting that NH_4_^+^ is a key pathway for DDN transfer to non-diazotrophic plankton [80]. Similar techniques applied to temperate waters of the Baltic Sea showed that the filamentous diazotroph *Aphanizomenon* transfers its DDN to the diatom *Chaetoceros* sp. and autotrophic and heterotrophic picoplankton within a few hours [84].

Higher in the food web, diazotrophic cyanobacteria have long been regarded as a bottleneck for the transfer of organic matter into zooplankton and upper trophic levels [85] due to their toxicity and poor nutritional quality [86], but recent observations challenge this prevailing view. Evidence of DDN in zooplankton comes from natural ^15^N isotopic measurements on zooplankton and the use of two-source N isotope mixing models, which estimated that DDN contributes to ~25% of the zooplankton N biomass in the Baltic Sea [87], 30%–40% in the tropical Atlantic [88, 89], and 67%–75% in the subtropical Pacific [90]. Direct observations, grazing experiments, and *nifH* detection in copepods’ full-guts demonstrate copepod grazing on *Trichodesmium* [72, 73], UCYN-A [75, 91], UCYN-B [75], UCYN-C [76], *Richelia* [75, 76] and *Aphanizomenon* [87]. Ciliates, mixotrophic dinoflagellates and coccolithophores graze on *Crocosphaera* (UCYN-B) [92], and the crown-of-thorns starfish completes their larval phase by feeding only on *Trichodesmium* [93]. Finally, ^15^N-labelling experiments confirmed that diazotrophs provide a direct source of N that supports zooplankton metabolism [76, 82, 84, 87, 94]. The DDN transfer efficiency appears species-specific as shown with DDN from UCYN seems to be more efficiently transferred to the food web than that of *Trichodesmium* [76, 82]. Collectively, these studies prove that diazotrophs support zooplankton. However, none of them have looked into DDN transfer into fecal pellets, despite the fact that pellets rapidly sink (100- > 1000 m d^−1^ [95]) and play a major role in OC export to the deep ocean [96] (see section 2) Table 1.

### (2) Role of diazotrophs in the biological carbon pump

The biological carbon pump (BCP) is the process by which CO_2_ is converted to OC through photosynthesis by phytoplankton in the surface ocean, exported through sinking particles and aggregates of different nature (e.g. dead organisms, fecal pellets), and finally sequestered in the deep ocean. The strength of the pump is controlled, in large part, by the rate of inorganic fixed N (notably nitrate, NO_3_^−^) resupply to the sunlit ocean (euphotic zone) [97]. However, nitrate is lacking in ∼60% of the ocean [1, 98], including areas with very low iron and, in some cases, phosphate concentrations [99].

In these expansive nutrient limited (oligotrophic) surface ocean regions, diazotrophs provide an alternative source of N through biological fixation of N_2_. This new N helps to maintain ocean fertility, promotes CO_2_ fixation into OC by phytoplankton, and in turn, sustains the food web and the BCP through the so called N_2_-primed prokaryotic C pump [2]. N_2_ fixation by diazotrophs provides the largest external source of N to the global ocean [46], yet the magnitude and pathways by which this diazotroph-derived organic carbon is exported to depth remain poorly constrained. This uncertainty hampers our ability to make robust predictions about the role of the ocean in CO₂ sequestration and its capacity to mitigate climate change, both now and in the future.

## Geochemical budgets

Presently, the role of diazotrophs on export production is mostly assessed through geochemical δ^15^N budgets. Budgets compare the distinct δ^15^N signature of the two dominant sources of new N to surface waters, subsurface NO_3_^−^ and newly fixed N, with the δ^15^N of the export flux, and provide an integrative measure of the relative contributions of both sources to export production [100, 101]. These budgets report that N_2_ fixation accounts for ~25%–50% of export production in the subtropical North Pacific (Station ALOHA, Hawaii) [102–104], ~10% in the subtropical North Atlantic (BATS) [101], and 50%–80% in the subtropical South Pacific [105, 106]. Such geochemical tools are extremely useful as they revealed that diazotrophs significantly impact OC export. However, interpreting isotopic signatures is complex. Alternative sources of fixed N, such as atmospheric deposition, can have a δ^15^N signature similar to N₂ fixation [107]. Additionally, potential under-collection of OC export by sediment traps, and temporal variations in the δ^15^N signature of nitrate (NO₃^−^) supplied to the euphotic zone introduce biases in isotopic N budgets [105]. Furthermore, isotopic signatures do not provide information on how the diazotroph community composition in the sunlit ocean influences the OC export efficiency (e-ratio = OC export/OC produced by primary production). Moreover, this method does not discriminate if diazotrophs are exported themselves (direct export) or if they sustained primary and secondary production that was subsequently exported (indirect export). Despite diazotrophs contribute to the BCP, their role and quantitative impact in shaping the export flux is poorly (not) considered in global climate models (see section below) due to large knowledge gaps on export pathways and sinking particle dynamics.

**Figure 3:**
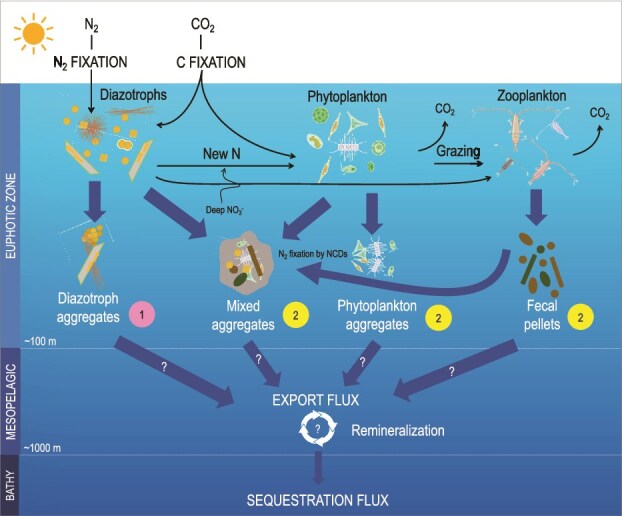
A schematic illustration of the possible export pathways of diazotrophs to the deep ocean. First, CO_2_ is photosynthetically fixed by diazotrophs into OC in the euphotic zone. By fixing N_2_, diazotrophs provide new N, promoting additional CO_2_ fixation by phytoplankton, which is in turn grazed by zooplankton, both producing diazotroph-derived OC. A fraction of this diazotroph-derived OC is exported to the mesopelagic zone by gravitational settling of (i) diazotrophs, (ii) and/or various types of aggregates resulting from phytoplankton and zooplankton partly derived from diazotrophy, on which NCDs may actively fix N_2_. In the mesopelagic, remineralization driven by bacteria and zooplankton recycle part of the sinking OC.

Diazotrophs are exported via two main pathways: (i) directly, through their own gravitational settling (Pathway 1, Fig. 3); and (ii) indirectly, after DDN is transferred to non-diazotrophic phytoplankton, zooplankton, or bacteria, which are then exported as aggregates and fecal pellets (Pathway 2, Fig. 3). Given these multiple export pathways, the resulting sinking particles can be diazotrophs themselves (single cells/filaments or diazotroph aggregates), phytoplankton derived from diazotrophy, zooplankton, detritus, fecal pellets, or a mixture of the above with particle sizes ranging from a few μm to several cm [95, 108]. Deciphering these pathways and their contribution to the BCP and carbon sequestration remains, to date, a great challenge. Below is a summary of our current knowledge, as well as the gaps we have identified and the avenues to be explored to further understand these export pathways.

### Direct export of diazotrophs to the deep ocean

Diazotrophs vary in size, morphology, and density. Some are free-living, unicellular and small (1–8 μm), whereas others, such as *Trichodesmium*, are filamentous and can form large-size colonies (>100–1000 μm). In addition, some diazotrophs live in symbioses with calcified (UCYN-A) or silicified eukaryotes (DDAs). These biominerals increase the particle density and provide ballast that potentially facilitates effective transport of the symbioses to the deep ocean. As a result, the presence of various diazotrophs in surface waters may result in dramatically different OC export fluxes. Yet, only a few field observations have to date related individual diazotroph groups to the magnitude of downward particles fluxes.

Until recently, much of our knowledge was based on cyanobacterial diazotrophs living in symbiosis with large diatoms (DDAs), which play an important role in OC export [109–111], and contribute annually to pulsed events of export flux during late summer at Station ALOHA [109]. The prevailing view within the scientific community has long been that, aside from DDAs, diazotrophs do not sink below the euphotic zone. This view was primarily based on two assumptions: (i) *Trichodesmium*, one of the major contributors to global N_2_ fixation [5, 112] possessed gas vesicles that confer buoyancy and prevent sinking into the deep ocean [113]. As a result, these colonies seldom reach depth, and their predominant remineralization is thought to occur in the upper ocean through surface cell autolysis [35, 114]; (ii) UCYN, the most abundant diazotrophs in the ocean [112] have long been considered to be too small (1–8 μm) to sink, or to do so at very low velocities [115], thereby favoring rapid remineralization in surface waters. The calcified form of the coccolithophore *Braarudosphaera bigelowii* [116], the host of UCYN-A2, has been detected in coastal sediment samples [117], suggesting their potential to sink, but direct evidence of UCYN-A or UCYN-B export was lacking, until recently [40].

Our understanding has gradually shifted with growing evidence of *Trichodesmium*, UCYN, and NCDs captured in sediment traps, not only just below the euphotic zone [31, 118], but as deep as 3–4000 m in the tropical Atlantic, Pacific and Indian Oceans [119–121]. More recently, the combined deployment of surface-tethered drifting sediment traps, Marine Snow Catcher, and Bottle-net sampling has enabled quantification of the direct export of diverse diazotrophs and provided insights into the fate of specific taxa within the mesopelagic ocean [40]. Observations showed that cyanobacterial and NCDs are massively exported down to 1000 m-depth, accounting for up to 80% of C at that depth. The Tara Oceans metagenomes collected in other ocean basins extended the scope of these results globally, and confirmed that gene sequences affiliated to surface inhabiting diazotrophs are also systematically present in mesopelagic waters [40].

These studies have challenged the common assumption that the fate of diazotroph-derived biomass is constrained to the surface ocean, but the processes that lead to the export of these organisms remain unknown. In the case of *Trichodesmium*, this is particularly intriguing given that natural populations are positively or neutrally buoyant thanks to their gas vesicles [113, 122]. This buoyancy is also the result of simultaneous daytime C and N_2_ fixation, that directly transfers energy from the photosystems to the nitrogenase enzyme, reducing glycogen ballast, and allowing *Trichodesmium* to remain at the surface [123]. Several hypotheses have been proposed to explain the occurrence of *Trichodesmium* in mesopelagic waters: (i) *Trichodesmium* colonies are known to select, collect and process dust particles to dissolve and utilize iron and phosphorus [124, 125]. The downside is that this mineral load can increase its density and hence the sinking velocity of *Trichodesmium* [123], although at typical oceanic dust fluxes, the dust load within *Trichodesmium* colonies does not seem to modify its sinking velocity [126]. (ii) *Trichodesmium* may also be ballasted by diatoms such as *Navicula, Nitzschia*, and *Cylindrotheca*, that are frequently found in association with colonies [49, 60]. Similarly, Zhang *et al.* [127] described calcifying amoebae, likely *Trichosphaerium micrum*, associated with *Trichodesmium*, that may enhance sinking. (iii) *Trichodesmium* colonies can also migrate vertically to exploit the deep phosphate stock [113, 122, 128]. According to this theory, they overcome their positive buoyancy by fixing C that results in carbohydrate ballasting. Walsby [129] observed that all gas vacuoles of *Trichodesmium erythraeum* collapse between 105 and 120 m, leading to a loss of buoyancy. Consequently, once *Trichodesmium* reaches this depth, it may become entrained in a persistent and irreversible downward trajectory. (iv) Additional experimental evidence demonstrates the existence and operation of programmed cell death (PCD) in *Trichodesmium* [130]. PCD induces gas vacuole loss, internal cellular degradation, and increased production of exopolymeric saccharides, resulting in an increase in the vertical flux of *Trichodesmium* [131, 132]. (v) Finally, physical processes may also enhance the transport of *Trichodesmium* to the deep ocean. In the North Pacific subtropical gyre, elevated *Trichodesmium* concentrations from a frontal region within the cyclonic-anticyclonic dipole are efficiently exported to the deep ocean [133]. According to these authors, surface downwelling in the frontal region between the two eddies, along with horizontal convergence, increase particulate OC export including that of *Trichodesmium*. The mechanisms discussed here are the only ones proposed to potentially drive the export of “floating” particles such as *Trichodesmium*, yet the processes underlying this export -as well as its quantification- remain to be conclusively demonstrated.

The case of UCYN is even more puzzling than that of *Trichodesmium*. A recent study on specific export turnover rates of diazotrophs (i.e. the fraction of surface diazotrophs exported out of the photic layer per day) showed that small UCYN are more efficiently exported relative to large *Trichodesmium* [40]. This is unexpected as individual cells have near-zero sinking velocities in seawater [115]. However, UCYN-B are frequently observed embedded in large (>50 μm) organic aggregates or organized into clusters of tens to hundreds of cells bound by an extracellular matrix, likely facilitating their export [81]. These observations align with experimental results from 55 000-L mesocosms in the New Caledonian lagoon, where a bloom of UCYN-C was efficiently exported via aggregation processes of small cells (~6 μm) into progressively larger particles with depth, reaching sizes of 100–500 μm in sediment traps [81].

NCDs also contribute to the direct export of diazotroph-derived biomass. Like UCYN, NCDs are individually too small to sink, but they are often associated with larger particles that exhibit significant sinking velocities, likely facilitating their transport to the deep ocean. Modeling and *in situ* studies suggest that NCDs actively fix N₂ at depth [20, 71], which may influence particle degradation during sinking, potentially affecting both the magnitude and quality of exported organic C.

### Sinking velocities and remineralization of diazotrophs

Although current evidence indicates that diazotrophs of diverse sizes, morphologies, and lifestyles have the potential to sink directly below the photic layer, numerous questions remain unresolved. As the OC flux declines by ~90% between 100 m and 1000 m [134], the presence of high quantities of diazotrophs at 1000 m suggests that they sink fast enough to escape short-term remineralization. However, sinking velocities of diazotrophs remain scarce. Bar-Zeev *et al.* [131] first reported that *Trichodesmium* aggregates from culture sank at ~200 m d^−1^ in an experimental column after stimulating a bloom and inducing PCD. By collecting natural *Trichodesmium* colonies at 100–250 m depth using a Marine Snow Catcher, Sargent [135] reported sinking velocities of 62 ± 40 m d^−1^, ranging from 12–120 m d^−1^. Ababou *et al.* [136] simulated the fall of cultured *Trichodesmium* in rolling tanks and reported comparable sinking velocities, averaging 92 ± 37 m d^−1^.

Data on UCYN sinking velocities are even rarer. Low sinking velocities of 0.071 m d^−1^ were initially reported for individual UCYN-B cells [115]. However, subsequent evidence indicates that UCYNs can sink more efficiently when incorporated into cell aggregates bound by TEP [40, 81]. Accordingly, experiments from rolling tanks estimated sinking velocities of 408 ± 172 m d^−1^ for UCYN-B aggregates and 102 ± 54 m d^−1^ for UCYN-C [136]. Given the scarcity of available measurements, further studies are needed to more accurately constrain diazotroph sinking velocities (Table 1).

Data on remineralization of diazotrophs are also scarce. The presence of intact [40] and even actively fixing *Trichodesmium* colonies down to 1000 m [137] remains intriguing, and suggests that *Trichodesmium* sink fast and escape remineralization. These findings contradict the common assertion that they are remineralized in the euphotic layer [35, 114]. Only one study reports results from a 10-day rolling tank experiment, simulating the fall of *Trichodesmium* aggregates down to 1000 m (based on a sinking velocity of ~100 m d^−1^ [136]). Under the experimental conditions (darkness, 20°C), 33% of the particulate OC and 36% of the particulate organic N derived from *Trichodesmium* remains intact at the end of the experiment, suggesting incomplete microbial remineralization at 1000 m (simulated depth) [138].

Further *in situ* studies are needed to confirm these first estimates. *Trichodesmium* filaments and colonies are quite large (>100 μm) and blooms in the subtropical ocean generate significant C biomass at the surface (850–26 000 mg C m^−2^ [40, 139]), potentially creating oasis in the otherwise oligotrophic ocean. It is therefore important to determine whether this surface-produced C has the potential to be sequestered in the deep ocean (>1000 m), in what proportion, and the factors that influence it (i.e. age of bloom, cause of demise, bacterial assemblage associated with the aggregates, nutrient and temperature conditions). It is also crucial to investigate the remineralization of other diazotrophs, particularly UCYN aggregates, which are large, dense, and frequently observed in the mesopelagic zone [40, 139] (Table 1).

### Indirect export of diazotrophs to the deep ocean

Diazotrophs are also exported through secondary pathways. In seawater, diazotrophs release 10–50% of recently fixed N_2_ (referred to as DDN) as NH_4_^+^ and dissolved organic N (DON) [140–142]. This DDN is potentially available for assimilation by the surrounding planktonic communities (see above), supporting their growth and leading a potential secondary (indirect) export pathway of diazotroph-derived OC [143].

To assess how much DDN potentially fuels secondary export, we need to quantify the DDN transfer to non-diazotrophic plankton, and to identify the communities benefiting from it. Techniques combining ^15^N_2_ isotopic labelling [60], cell sorting by flow cytometry and single-cell ^15^N isotopic analyses using nanoSIMS have been used to trace the DDN in surrounding phytoplankton and bacteria (see review in section 1), and show that ballasted organisms such as diatoms benefit from the DDN and have the potential to contribute to this indirect export of diazotroph-derived biomass.

Secondary export pathways cannot be studied without accounting for the role of zooplankton. DDN is efficiently transferred to zooplankton [76], which package diazotroph organic matter into fecal pellets. Pellets in turn, sink rapidly [95] and might play a major role in DDN export to the deep ocean. As diazotrophs are more commonly grazed than previously thought (see section 1) they can be a direct source of N for zooplankton metabolism. However, to our knowledge, no study has yet demonstrated the transfer of DDN into fecal pellets, despite their rapid sinking rates (100- > 1000 m d^−1^ [95]) and their well-established role in exporting organic carbon to the deep ocean [96]. Initial analyses of the composition of the sinking OC flux in polyacrylamide gel-filled traps in a hot spot of N_2_ fixation (South Pacific), revealed that fecal aggregates dominate the flux, accounting for >50% of the sinking OC flux [139]. As N isotope budgets show that export is mainly supported by diazotrophy in this region [105], these results suggest that the DDN is efficiently transferred through the food web to zooplankton and fecal pellets prior to export [139]. These results indicate that indirect export would be the main export vector for diazotroph-derived biomass, but further studies in several ocean basins at different seasons are required to better understand how surface plankton communities shape export pathways Table 1.

### Towards a better representation of diazotroph-mediated export in biogeochemical models?

The scientific community greatly progressed during the past few decades in describing the diversity of diazotrophs, their ecological niche, their environmental regulatory factors, and their role in biogeochemical cycles. Moreover, the representation of diazotrophs, long only implicit in biogeochemical models, is greatly improving [29, 30, 144–146]. Models are now integrating explicit representations of different groups with distinct physiological characteristics and potential varying responses to climate change (e.g. [29, 147–149]). N₂ fixation is likely to play a key role in future ocean net primary production [30, 145, 149], although uncertainties remain regarding diazotroph physiological responses [29] and iron availability [148, 149] under future climate scenarios.

Overall, it is clear that N_2_ fixation will likely play an important role in net primary production in the future ocean [30, 145, 149], although substantial uncertainties remain regarding the physiological responses of different groups of diazotrophs [29], and different iron conditions [148, 150] to future climate scenarios.

Within the limits of our current understanding, the potential role of diazotrophs in the export and storage of OC is still poorly accounted for in biogeochemical models [151]. In models where diazotrophy is implicitly represented, its potential role in organic matter export is captured indirectly through the supply of new N to the planktonic communities of the ocean surface layer. This assumption enhances new primary production and, consequently, potential export, which in this framework corresponds to an indirect export pathway. By contrast, the direct export of carbon derived from diazotrophs is not represented, as diazotrophs are typically assigned to non-sinking particle pools. In models where diazotrophs are represented explicitly (e.g. [29, 147–149]), it remains impossible to capture the differential fate of individual functional groups in the deep ocean. Addressing major knowledge gaps -such as quantifying the transfer of DDN to other planktonic groups, determining sinking velocities, and constraining remineralization rates- is essential for improving model accuracy and better representing diazotroph contributions to biogeochemical cycles (Table 1).

Apart from diazotrophs, processes that drive OC export to the deep ocean are already very complex, and export fluxes are poorly constrained in current models [152, 153]. Consequently, the most recent global climate models, (e.g. the Intergovernmental Panel on Climate Change, IPCC), report very different projections of export flux by 2100 (+1.8 to −41%) [153]. Because it is not feasible to represent every mechanistic parameterization of each possible process in models due to computational constraints, choices must be made regarding which processes are most critical. To improve the representation of the links between diazotrophy and export, we specifically recommend: (i) identifying which plankton functional types or size fractions benefit from DDN in the sunlit ocean; (ii) characterizing the size, density, and shape of diazotroph-derived particles; (iii) determining their sinking velocities; (iv) quantifying remineralization rates along depth and temperature gradients; and (v) assessing whether these particles are consumed and fragmented by zooplankton, and how this influences particle characteristics and remineralization.

The vast extent of the oceans, combined with the high spatial and temporal variability of marine ecosystems, makes highly resolved sampling and observation programs extremely challenging, particularly with regard to OC export and, more specifically, diazotroph-mediated export. The development and increasing use of autonomous platforms such as moorings, floats, and gliders are providing a growing number of valuable observations. However, concurrent measurements of surface ocean characteristics related to phytoplankton (e.g. community composition, productivity) and of the transformations undergone by sinking particles (mortality, aggregation, fragmentation, remineralization) remain scarce. To improve models, it is essential to better link surface-layer processes with the resulting quantity and quality of organic matter exported to depth. Fully understanding the mechanisms governing the BCP and predicting its future evolution will require overcoming technological limitations and deploying long-term, high-frequency (hourly to daily) autonomous observational systems capable of simultaneously monitoring both surface and deep ocean processes.

## Conclusions and summary of future directions

Over the last three decades, our perception of marine diazotrophy has shifted profoundly. Once considered a marginal process restricted to a handful of filamentous cyanobacteria in tropical waters, N₂ fixation is now recognized as a globally distributed phenomenon carried out by a broad range of cyanobacterial and non-cyanobacterial diazotrophs across diverse marine habitats. These organisms supply an essential source of new N to oligotrophic ecosystems, thereby sustaining primary productivity and shaping trophic interactions. Beyond their role as N providers, diazotrophs contribute to the BCP through multiple pathways: directly, via the export of their own biomass, and indirectly, by fueling phytoplankton, zooplankton, and higher trophic levels that ultimately drive carbon export to depth.

Despite substantial advances, key challenges persist Table 1. The efficiency of DDN transfer through food webs, the fate of their biomass during export, and the sinking velocities and remineralization dynamics of different taxa remain poorly constrained. Equally, the role of zooplankton and the contribution of fecal pellet-mediated export are still largely unexplored. These uncertainties limit our ability to quantify the true role of diazotrophs in C cycling and hinder their integration into global biogeochemical and climate models.

Addressing knowledge gaps will require linking molecular and isotopic approaches with long-term autonomous observations that capture processes from the surface to the deep ocean, and embedding mechanistic insights into models that explicitly represent the diversity of diazotroph functional groups. Such efforts are essential to anticipate how diazotrophy will respond to a changing climate and to better constrain its role in sustaining marine productivity and regulating long-term C sequestration.

### Note on methods

The *nifH* sequences from size fractionated samples were recruited from metagenomic reads [36]. The data set is available here: https://static-content.springer.com/esm/art%3A10.1038%2Fs41467–021-24 299-y/MediaObjects/41467_2021_24 299_MOESM11_ESM.xlsx. In each size fraction (0.8–5 μm, 5–20 μm, 20–180 μm and 180–2000 μm), sequences were normalized by recA reads, used as a proxy of bacterial abundances. The different *nifH* reads were clustered into the following taxonomic assignations: *Trichodesmium, Crocosphaera, Richelia*, UCYN-A1, UCYN-A2, *Planctomycetes* and *Proteobacteria*. For each size fraction, we considered only the diazotroph groups detected in more than seven samples. The size fractioned data of the V4 region of the 18S rDNA genes from amplicon sequencing was retrieved from Mahé *et al.* (2022), available here: https://zenodo.org/records/6794519. We kept only the most abundant ASVs (more than 5000 reads in total for each size fraction). Pearson correlation between diazotrophs and 18S ASVs were tested individually for each size fraction on clr-transformed absolute abundances for 18S ASVs and on log10 transformed relative abundances of *nifH* groups. Correlations were calculated and significance (Pearson correlation, *P <* .0001) tested using the “rcorr” function from the “Hmisc” R package (R version 4.4.2). The produced interactome was then simplified by removing the size fraction information. When multiple identical diazotroph-ASVs correlations were detected in different size fractions, we kept the correlation with highest correlation coefficient. Only the significant correlations (*r* > 0.39, *P <* .0001) were kept. Data were arranged as “igraph” object using the “igraph” package before being plotted using the Gephi software (v 0.10.1). The interactome is available in Supplementary material (Table S2). Readers should note that co-occurrence analyses reveal patterns, not causal links.

## Supplementary Material

wraf291_Supplemental_Files

## Data Availability

The dataset analyzed during the current study are available in the Zenodo repository, https://zenodo.org/records/6794519.
